# Validation of a Novel Three-Dimensional (*3D Fusion*) Gross Sampling Protocol for Clear Cell Renal Cell Carcinoma to Overcome Intratumoral Heterogeneity: The Meet-Uro 18 Study

**DOI:** 10.3390/jpm12050727

**Published:** 2022-04-30

**Authors:** Matteo Brunelli, Guido Martignoni, Giorgio Malpeli, Alessandro Volpe, Luca Cima, Maria Rosaria Raspollini, Mattia Barbareschi, Alessandro Tafuri, Giulia Masi, Luisa Barzon, Serena Ammendola, Manuela Villanova, Maria Angela Cerruto, Michele Milella, Sebastiano Buti, Melissa Bersanelli, Giuseppe Fornarini, Sara Elena Rebuzzi, Valerio Gaetano Vellone, Gabriele Gaggero, Giuseppe Procopio, Elena Verzoni, Sergio Bracarda, Martina Fanelli, Roberto Sabbatini, Rodolfo Passalacqua, Bruno Perrucci, Maria Olga Giganti, Maddalena Donini, Stefano Panni, Marcello Tucci, Veronica Prati, Cinzia Ortega, Anna Caliò, Albino Eccher, Filippo Alongi, Giovanni Pappagallo, Roberto Iacovelli, Alessandra Mosca, Paolo Umari, Ilaria Montagnani, Stefano Gobbo, Francesco Atzori, Enrico Munari, Marco Maruzzo, Umberto Basso, Francesco Pierconti, Carlo Patriarca, Piergiuseppe Colombo, Alberto Lapini, Giario Conti, Roberto Salvioni, Enrico Bollito, Andrea Cossarizza, Francesco Massari, Mimma Rizzo, Renato Franco, Federica Zito-Marino, Yoseba Aberasturi Plata, Francesca Galuppini, Marta Sbaraglia, Matteo Fassan, Angelo Paolo Dei Tos, Maurizio Colecchia, Holger Moch, Maurizio Scaltriti, Camillo Porta, Brett Delahunt, Gianluca Giannarini, Roberto Bortolus, Pasquale Rescigno, Giuseppe Luigi Banna, Alessio Signori, Miguel Angel Llaja Obispo, Roberto Perris, Alessandro Antonelli

**Affiliations:** 1 Pathology Unit, Department of Diagnostics and Public Health, University and Hospital Trust of Verona, 37134 Verona, Italy; matteo.brunelli@univr.it (M.B.); guido.martignoni@univr.it (G.M.); serena.ammendola88@gmail.com (S.A.); manuela.villanova@univr.it (M.V.); anna.calio@univr.it (A.C.); albino.eccher@aovr.veneto.it (A.E.); 2 FISH Lab, Renal Cancer Center Room, Department of Diagnostics and Public Health, University and Hospital Trust of Verona, 37134 Verona, Italy; 3 Pathology Unit, Pederzoli Hospital, Peschiera del Garda, 37019 Peschiera, Italy; 4 Department of Surgery, Dentistry, Paediatrics and Gynaecology, University of Verona, 37134 Verona, Italy; 5 Division of Urology, Maggiore della Carità Hospital, 28100 Novara, Italy; alessandro.volpe@med.unipmn.it; 6 Pathology Unit, Department of Clinical Services, Santa Chiara Hospital, 38100 Trento, Italy; lucacima85@gmail.com (L.C.); mattia.barbareschi@apss.tn.it (M.B.); 7 Histopathology and Molecular Diagnostics, University Hospital Careggi, 50100 Firenze, Italy; mariarosaria.raspollini@unifi.it (M.R.R.); paolo.umari@med.uniupo.it (P.U.); i.montagnani@libero.it (I.M.); 8 Division of Urology, Department of Surgery, Dentistry, Paediatrics and Gynaecology, University and Hospital Trust of Verona, 37134 Verona, Italy; alessandro.tafuri@univr.it (A.T.); mariaangela.cerruto@univr.it (M.A.C.); alessandro.antonelli@univr.it (A.A.); 9 Department of Molecular Medicine, University of Padua, 35100 Padova, Italy; giulia.masi@unipd.it (G.M.); luisa.barzon@unipd.it (L.B.); 10 Division of Oncology, University and Hospital Trust of Verona, 37134 Verona, Italy; michele.milella@aovr.veneto.it; 11 Division of Oncology, University and Hospital Trust of Parma, 43100 Parma, Italy; sbuti@ao.pr.it (S.B.); bersamel@libero.it (M.B.); 12 Division of Oncology, San Martino Hospital, 16100 Genova, Italy; giuseppe.fornarini@hsanmartino.it (G.F.); saraelena89@hotmail.it (S.E.R.); 13 Pathology Unit, San Martino Hospital, 16100 Genova, Italy; valerio.vellone@unige.it (V.G.V.); gabriele.gaggero@hsanmartino.it (G.G.); 14 Division of Oncology, IRCCS Foundation, Istituto Nazionale dei Tumori di Milano, 20133 Milano, Italy; giuseppe.procopio@istitutotumori.mi.it (G.P.); elena.verzoni@istitutotumori.mi.it (E.V.); 15 Division of Oncology, Santa Maria Hospital, 05100 Terni, Italy; s.bracarda@aospterni.it; 16 Division of Oncology, Department of Oncology, Hematology & Respiratory Diseases, University of Modena & Reggio Emilia, 41121 Modena, Italy; martinafun89@gmail.com (M.F.); roberto.sabbatini@unimore.it (R.S.); 17 Division of Oncology, Hospital Trust of Cremona, 26100 Cremona, Italy; r.passalacqua@asst-cremona.it (R.P.); brunoperrucci@yahoo.it (B.P.); m.giganti@asst-cremona.it (M.O.G.); maddalenadonini@gmail.com (M.D.); s.panni@asst-cremona.it (S.P.); 18 Division of Oncology, Cardinal Massaia Hospital, 14100 Asti, Italy; mtucci@asl.at.it; 19 Division of Oncology, Institute for Cancer Research and Treatment, Asl Cn2 Alba-Brà, 12042 Cuneo, Italy; veronica.prati@ircc.it (V.P.); cinzia.ortega@ircc.it (C.O.); 20 Advanced Radiation Oncology Department, IRCCS Ospedale Sacro Cuore Don Calabria, Negrar, 37024 Verona, Italy; filippo.alongi@sacrocuore.it; 21 Indipendent Researcher, Clinical Epidemiologist, Silea, 31057 Treviso, Italy; giovanni.pappagallo@tin.it; 22 Policlinico Universitario Fondazione Andrea Gemelli, 00168 Roma, Italy; roberto.iacovelli@policlinicogemelli.it; 23 Oncology, Candiolo Cancer Institute, IRCCS-FPO, 10060 Torino, Italy; alessandramosca25@yahoo.it; 24 Department of Translation Medicine, Pathology Unit, University of Ferrara, 44100 Ferrara, Italy; gobo79@gmail.com; 25 Oncology Unit, University and Hospital Trust of Cagliari, 09100 Cagliari, Italy; francescoatzori74@yahoo.it; 26 Pathology Unit, Department of Molecular and Translational Medicine, University of Brescia, 25100 Brescia, Italy; enrico_munari@yahoo.it; 27 Oncology Unit, Oncology Department, Istituto Oncologico Veneto (IOV), IRCCS, 35128 Padua, Italy; marco.maruzzo@iov.veneto.it (M.M.); umberto.basso@iov.veneto.it (U.B.); 28 Division of Pathology, Policlinico Universitario Fondazione Agostino Gemelli, 00168 Roma, Italy; francesco.pierconti@policlinicogemelli.it; 29 Pathology Unit, Department of Clinical Services, Sant’Anna Hospital, 22100 Como, Italy; carlo.patriarca@hsacomo.org; 30 Department of Pathology, Department of Biomedical Sciences, Humanitas University, IRCCS Humanitas Clinical and Research Hospital, Pieve Emanuele, 20089 Milan, Italy; piergiuseppe.colombo@humanitas.it; 31 Division of Urology, University Hospital Careggi, 50100 Firenze, Italy; lapinial@gmail.com; 32 Division of Urology, Sant’Anna Hospital, 22100 Como, Italy; giario.conti@asst-lariana.it; 33 Department of Urology, Fondazione IRCCS Istituto Nazionale dei Tumori di Milano, 20133 Milano, Italy; roberto.salvioni@istitutotumori.mi.it; 34 Department of Pathology, San Luigi Gonzaga Hospital, University of Turin, 10122 Torino, Italy; enrico.bollito@sanluigi.piemonte.it; 35 Department of Medical and Surgical Sciences for Children and Adults, University of Modena & Reggio Emilia, 41121 Modena, Italy; andrea.cossarizza@unimore.it; 36 Medical Oncology, University and Hospital Trust of Bologna, 40138 Bologna, Italy; francesco.massari@aosp.bo.it; 37 Division of Translational Oncology, IRCCS Istituti Clinici Scientifici ‘Maugeri’, 27100 Pavia, Italy; rizzo.mimma@gmail.com; 38 Pathology Unit, Department of Mental and Physical Health and Preventive Medicine, University of Campania ‘Luigi Vanvitelli’, 81100 Caserta, Italy; renato.franco@unicampania.it (R.F.); federica.zitomarino@unicampania.it (F.Z.-M.); 39 Department of Pathology, Cruces University Hospital, University of the Basque Country (UPV/EHU), 48900 Barakaldo, Spain; aberasturiy@gmail.com; 40 Department of Pathology, University and Hospital Trust of Padua, 35128 Padova, Italy; francesca.galuppini@unipd.it (F.G.); marta.sbaraglia@aopd.veneto.it (M.S.); matteo.fassan@unipd.it (M.F.); angelo.deitos@unipd.it (A.P.D.T.); 41 Department of Pathology, Uropathology Unit, Vita-Salute San Raffaele University, IRCCS San Raffaele, 20132 Milano, Italy; colecchia.maurizio@hsr.it; 42 Department of Pathology and Molecular Pathology, University Hospital Zurich, 8006 Zurich, Switzerland; holger.moch@usz.ch; 43 AstraZeneca, New York, NY 10005, USA; maurizio.scaltriti@astrazeneca.com; 44 Department of Biomedical Sciences and Human Oncology, University of Bari ‘A. Moro’, 70121 Bari, Italy; camillo.porta@uniba.it; 45 Division of Medical Oncology, Hospital Trust of Bari, 70121 Bari, Italy; 46 Department of Pathology and Molecular Medicine, Wellington School of Medicine and Health Sciences, University of Otago, 3743 Wellington, New Zealand; brett.delahunt@otago.ac.nz; 47 Urology Unit, “Santa Maria della Misericordia”, University of Udine, 33100 Udine, Italy; gianluca.giannarini@hotmail.it; 48 Department of Radiotherapy, CRO, 33081 Aviano, Italy; rbortolus@cro.it; 49 Candiolo Cancer Institute, FPO-IRCCS, Candiolo, 10060 Turin, Italy; pasquale.rescigno@ircc.it (P.R.); giuseppe.banna@ircc.it (G.L.B.); 50 Department of Health Sciences (DISSAL), Section of Biostatistics, University of Genova, 16100 Genova, Italy; alessio.signori.unige@gmail.com; 51 Medical Oncology Unit 1, IRCCS Ospedale Policlinico San Martino, 16100 Genova, Italy; miguelangel.llajaobispo@hsanmartino.it; 52 Department of Biosciences, COMT-Centre for Molecular and Translational Oncology, University of Parma, 43124 Parma, Italy; roberto.perris@unipr.it

**Keywords:** clear cell renal cell carcinoma, tumor sampling, intratumoral heterogeneity, angiogenesis, immunity, immunohistochemistry

## Abstract

We aimed to overcome intratumoral heterogeneity in clear cell renal cell carcinoma (clearRCC). One hundred cases of clearRCC were sampled. First, usual standard sampling was applied (1 block/cm of tumor); second, the whole tumor was sampled, and 0.6 mm cores were taken from each block to construct a tissue microarray; third, the residual tissue, mapped by taking pieces 0.5 × 0.5 cm, reconstructed the entire tumor mass. Precisely, six randomly derived pieces of tissues were placed in each cassette, with the number of cassettes being based on the diameter of the tumor (called multisite 3D fusion). Angiogenic and immune markers were tested. Routine 5231 tissue blocks were obtained. Multisite 3D fusion sections showed pattern A, homogeneous high vascular density (10%), pattern B, homogeneous low vascular density (8%) and pattern C, heterogeneous angiogenic signatures (82%). PD-L1 expression was seen as diffuse (7%), low (33%) and absent (60%). Tumor-infiltrating CD8 scored high in 25% (pattern hot), low in 65% (pattern weak) and zero in 10% of cases (pattern desert). Grading was upgraded in 26% of cases (G3–G4), necrosis and sarcomatoid/rhabdoid characters were observed in, respectively, 11 and 7% of cases after 3D fusion (*p* = 0.03). CD8 and PD-L1 immune expressions were higher in the undifferentiated G4/rhabdoid/sarcomatoid clearRCC subtypes (*p* = 0.03). Again, 22% of cases were set to intermediate to high risk of clinical recurrence due to new morphological findings of all aggressive G4, sarcomatoid/rhabdoid features by using 3D fusion compared to standard methods (*p* = 0.04). In conclusion, we propose an easy-to-apply multisite 3D fusion sampling that negates bias due to tumor heterogeneity.

## 1. Introduction

It is a critical feature of pathology practice that samples selected from specimens of tumors reflect their biological behavior. If sampling is not representative, the risk is that key diagnostic/prognostic features will be overlooked [1]. The development of tumor heterogeneity de novo or subsequent to therapy is an established feature of malignancies [2], and in the case of clear cell renal cell carcinoma (clearRCC), the existence of molecular heterogeneity is well recognized [3,4,5].

It is recommended for the sampling of renal malignancies that one block of tissue be taken per cm of tumor diameter [6,7], while molecular studies are often based upon a single tissue sample. Although an extensive amount of molecular information may be derived from a single fragment of tumor, the data are limited by the likelihood that they underestimate the entire picture.

Several studies have investigated the vascularity of clearRCC with varying results, and as a consequence, our understanding of the assessment of genetic signatures and patterns of angiogenesis for clearRCC is in evolution [8,9,10,11,12,13,14,15,16]. The advent of immunotherapy based upon immune checkpoint blockade, in addition to the development of drugs that target angiogenesis, has meant that it has become increasingly important to determine which patients with advanced disease are best treated by these adjuvant therapies [17,18,19,20,21,22,23,24,25,26]. In view of this, it is imperative that there is some certainty that sections taken from cases of clearRCC are representative of the tumor with prognostic parameters that will reflect outcome. Moreover, recent adjuvant therapies have been proposed and approved in locally advanced renal cancer, as pembrolizumab treatment has led to a significant improvement in disease-free survival compared with placebo after surgery among patients with kidney cancer who were at high risk for recurrence [27].

The Meet-Uro 18 is a group of Italian researchers committed to developing a retrospective observational study on the predictive and prognostic role of biomarkers in renal cancer. In this study, we developed a three-dimensional gross sampling methodology, which we designated *3D fusion*. This is designed to circumvent the apparent undersampling of renal tumors due to intrinsic heterogeneity. We utilized this methodology to investigate morphologic and immunohistochemical heterogeneity in clear cell RCC through an analysis of angiogenic and immune patterns.

## 2. Materials and Methods

### 2.1. Ethical Approval

This study was approved by the Institutional Review Board of the Department of Diagnostics and Public Health, University of Verona, in accordance with the Helsinki Declaration of 1975. Ethical Approval Number: PRIHTA2014-00000453. Informed consent was obtained from all subjects involved in the study.

### 2.2. Case Selection and Sampling Methodology

We recruited 100 patients with staging category ≥pT3a RCC treated by radical nephrectomy.

Gross examination of the specimens was performed by two specialist uropathologists (Matteo Brunelli and Guido Martignoni), and the tumors were photographed. Tumors were sectioned along the longitudinal axis at 0.5 cm intervals. Samples were taken for routine diagnostic purposes, and the remaining tissues were fixed in formalin and embedded in paraffin, with sections taken from each block.

**Figure 1:**
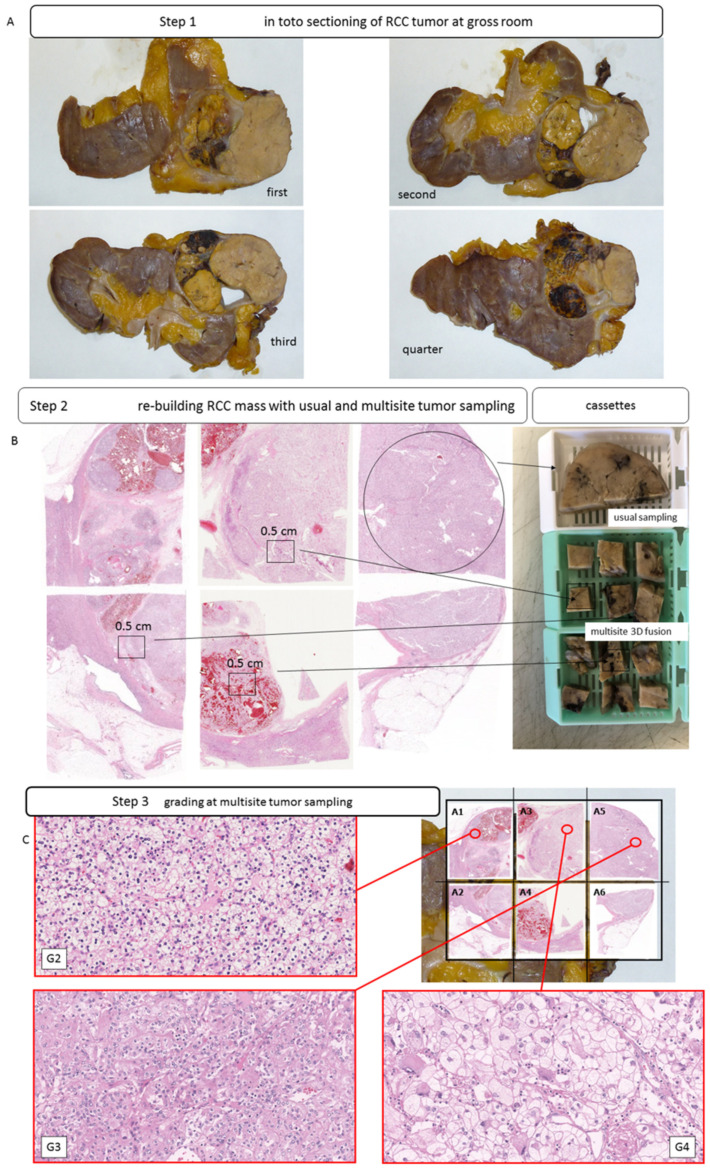
Intratumoral heterogeneity in clear cell RCCs. Gross images of different slices of clear cell RCCs with heterogenous macroscopic appearance (**A**); usual block (white cassette) and multisite sampling with 6 pieces of tumor tissue per single block (green cassette) and *3D fusion* multisite reconstruction (**B**,**C**); H&E staining (**C**).

Three sets of samples were obtained. In the first set, the tumor was sampled utilizing standard procedures with a single fragment of tissue (3 × 1.5 cm) taken per cm of tumor diameter, according to standard gross sampling recommendations [6,7]. The tumor was then sectioned in entirety, and a single punch was taken from the central portion of each section. These cores were then used to construct tissue microarrays (TMAs). The third method of sampling involved a novel *3D fusion* multisite tumor sampling protocol. For *3D fusion*, sampling was based on the size of the tumor. For each cm diameter of tumor six blocks of tissue, each measuring 0.5 × 0.5 cm, were randomly selected and placed in an individual cassette (steps shown in Figure 1A–C).

**Figure 2:**
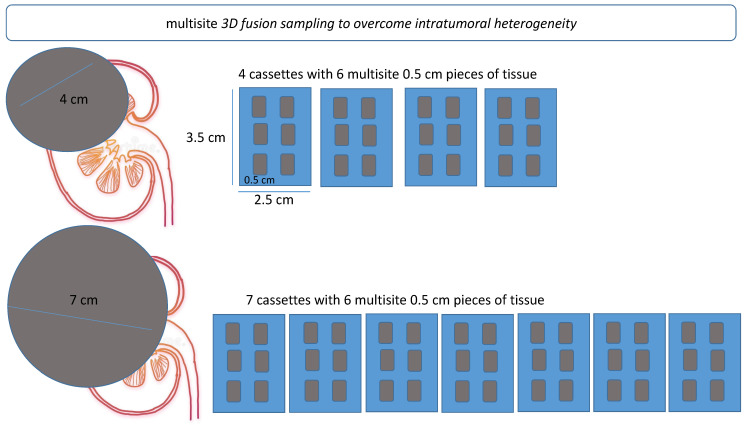
Schematic representation of multisite 3D fusion sampling procedure. Fusion sampling was based on the size of the tumor. For each cm diameter of tumor, six blocks of tissue, each measuring 0.5 × 0.5 cm, were randomly selected and placed in an individual cassette.

A representative cartoon of multisite *3D fusion* sampling is shown in Figure 2.

For *3D fusion*, sampling the blocks were embedded according to the site of origin in the kidney, with the larger sections and each section constituent smaller fragments being arranged from top to bottom and from left to right, thus representing multiple coronal sections of the kidney upon reconstruction. The site of origin of each tissue sample was mapped to produce a complete reconstruction of the tumor in three dimensions (Figure 2). Nine TMAs were built using the manual tissue arrayer MTA-1 (Beecher Instruments Inc., Madison, WI, USA). The section of each tissue core measured 0.6 mm in diameter, with an area of 0.28 mm^2^.

For each sampling method (routine, *3D fusion* multisite sampling and TMAs), 3 µm thick sections were cut and stained with routine hematoxylin and eosin (H&E) and examined by specialist uropathologists (Matteo Brunelli, Guido Martignoni, Anna Caliò).

### 2.3. TCGA Transcriptome Investigation

The publicly available dataset for Kidney Renal Clear Cell Carcinoma, PanCancer Atlas, the Cancer Genome Atlas (TCGA): https://www.cancer.gov/aboutnci/organization/ccg/research/structural-genomics/tcga) was accessed on 10 February 2022 through the cBioPortal [28,29,30]. The analysis of the genome-wide TCGA public molecular repository was undertaken with the aim of determining the profile of angiogenic and immune signatures for clearRCC.

### 2.4. Immunohistochemical Staining

For the evaluation of the vessel numbers and microvascular density (MVD), sections were stained using CD34 antibody (monoclonal, mouse, clone QBEnd/10, 1:1200 dilution, high temperature and pH 6 buffer antigen retrieval, Novocastra) and CD31 antibody (monoclonal, mouse, clone JC70A, 1:50, high temperature and pH 6 buffer antigen retrieval, Dako).

PD-L1 expression was evaluated by using two clones (E1L3N Cell Signaling and sp263 Ventana platform). Percentages of neoplastic cells were used for categorization between strong 2+ (≥50% of cells), low/weak 1+ (≥1 × <50% positive cells) and absence (score 0) of expression (<1%).

Tumor-infiltrating cells CD8^+^ were scored according to the number of CD8^+^ cells per mm^2^ after digital 25th and 75th percentiles of T, CD8^+^ cells. Interpretations were categorized for semantics as follows: hot/weak and desert forms.

### 2.5. Digital Slides Image Capture and Evaluation

Following immunohistochemical staining digital images from routine, *3D fusion* multisite and TMA sections were acquired using Grundium Ocus (Tampere, Finland) at 20× magnification and stored in a jpg file format. Digital slides were evaluated with ImageJ, an open-source program for image analysis and processing.

### 2.6. Statistical Analysis

The number of vessels defined as CD31- or CD34-positive structures (spots) were determined for each core. The digital results were compared to the microscopic count from paraffin sections. The mean, median, standard deviation (SD) and coefficient of variation (CV) were assessed. A cut-off of CV < 0.2 was utilized to define the vascular staining of a case as homogeneous. From this, the MVD, expressed as positive vessels/mm^2^, was assessed for every core.

The concordance index and Fisher’s test were applied to the comparison of the three methods with all clinical-pathological results. Cohen’s kappa statistics, as a measure of interrater agreement (manual count of vessels per tissue fragment, MVD values on single TMA tissue cores, tissues obtained from routine sampling and from *3D fusion* multisite sampling) were calculated by the use of Stata software version 16 (StataCorp). Correlations between all tested methods and all clinic-pathological findings were evaluated. A *p*-value less than 0.05 was considered statistically significant.

## 3. Results

### 3.1. Clinicopathological Characteristics of Patients

The series consisted of 69 males and 31 females (mean age: 66 years). Fifty-four tumors were located in the right kidney and 46 in the left kidney. pT staging category was pT3a for 68 cases, pT3b for 28 cases and pT4 for 6 cases. Utilizing the WHO/ISUP grading system for clearRCC [31], 9 tumors were grade 2, 61 were grade 3 and 30 were grade 4. Rhabdoid differentiation was present in nine cases, while eight cases showed sarcomatoid differentiation. The clinicopathological features are summarized in Table 1.

A total of 656 gross images were collected, representing the three-dimensional imaging of the tumors. The number of tissue blocks for each tumor ranged from 33 to 198 (mean: 53), with a total of 5231 tissue blocks being examined for the whole series. With the addition of the TMA samples, a total of 8324 tumor sections were examined for the various components of the study

### 3.2. TCGA Findings—CD31 and CD34 mRNA Levels in clearRCC

**Figure 3:**
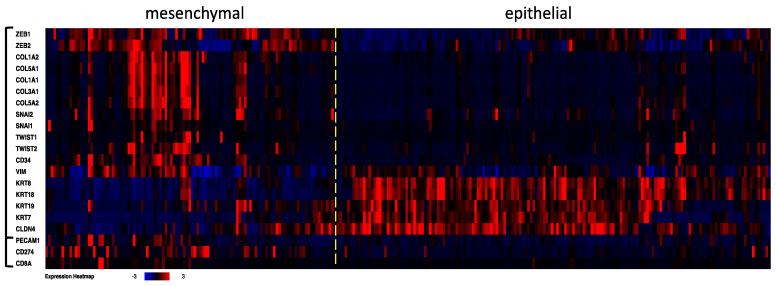
Transcriptome investigation in 512 clear cell RCC cases from TCGA (PanCancer Atlas study). The mRNA levels are z-scores relative to diploid samples (RNA Seq V2 RSEM). Clusterization procedure was applied to the first 18 genes, then the *PECAM1* (*CD31*), *CD274* (*PD-L1*) and *CD8A* mRNA levels were added to the established case list order. A dashed line was added to separate mesenchymal and epithelial clearRCC cases. The heat map shows angiogenesis and immune signatures in the clearRCC epithelial and mesenchymal subtypes. Higher levels of angiogenic markers *CD31* and *CD34* and of the immune markers *PD-L1* and *CD8A* come up predominantly in the mesenchymal subtype.

We evaluated the genome of the angiogenic and immunoexpression patterns in 512 clearRCC cases included in the Kidney Renal Clear Cell Carcinoma study (PanCancer Atlas, TCGA) and additionally focused on CD31 (*PECAM1*), *CD274* and *CD8A* expression. We established epithelial and mesenchymal (stem cell, undifferentiated) subtypes by the clustering of 18 genes, including the six classical transcriptional inhibitors and epithelial–mesenchymal transition markers *ZEB1*, *ZEB2*, *SNAI*, *SNAI2*, *TWIST1* and *TWIST2*, as well as CD34 and genes that discriminated “epithelial” and undifferentiated/mesenchymal subtypes in different cancer types [32] (Figure 3). *PECAM1* (CD31), *CD274* (PD-L1) and *CD8A* (CD8) were then added to the established case list in order to show the level of the three genes in the two clearRCC subtypes. Of the samples, about 40% were identified as having a mesenchymal (stem cell undifferentiated) genome, while about 60% had an “epithelial” genome, namely those cases expressing a very low level of EMT markers. *CD34*, *PECAM1*, *CD274* and *CD8A* genes were mostly overexpressed in the undifferentiated/mesenchymal stem cell clearRCC subtype. CD31 and CD34 strongly correlated with *ZEB1* (q-value = 5.3 × 10^−24^ and 4.8 × 10^−57^, respectively) and to lesser extent with *ZEB2* (q = 3.0 × 10^−5^ and 1.8 × 10^−13^, respectively) levels.

### 3.3. Immunohistochemical Findings

The expression of CD31 and CD34 in tissues obtained from routine sampling was compared with that obtained from *3D fusion* multisite sampling and TMA core samples (Table 2).

CD31 and CD34 immunoexpressions arose in all samples but variably and independently in terms of scoring in both undifferentiated and differentiated subtypes (*p* < 0.001). The digital vessel counts of the entire series showed a mean of 90 vessels/mm^2^, with a median of 60, an SD of 85 and a CV of 0.97 for CD31 and a mean of 113 vessels/mm^2^, with a median of 93, an SD of 93 and a CV of 0.82 for CD34. Manual light microscopic evaluation of the entire series produced a mean of 102 vessels/mm^2^, median of 82, SD of 81 and CV of 0.80 for CD31 and a mean of 99, median of 68, SD of 96 and CV of 0.95 for CD34.

**Figure 4:**
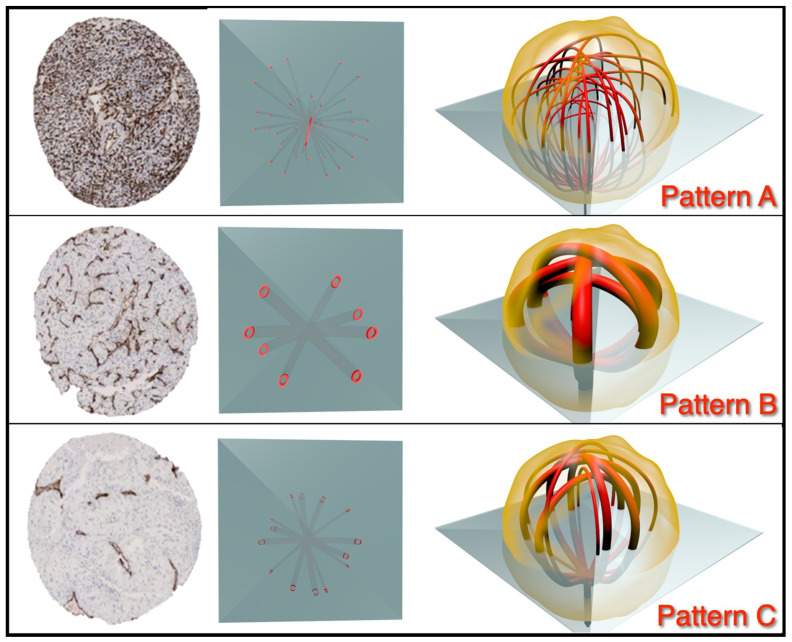
Classification of angiogenesis in clear cell RCCs after *3D fusion* multisite tumor sampling. Diagram showing categorization of pattern of angiogenesis: *pattern A,* characterized by homogeneous high level of angiogenesis; *pattern B*, characterized by homogeneous low level of angiogenesis; and *pattern C*, characterized by a mixture of patterns.

Utilizing the 3D fusion tumor samples, CD31 immunohistochemistry showed three patterns of angiogenesis (Figure 4): pattern A, homogeneous with high vascular density (mean CD31 density: 410 spots, SD: 208) (10% of cases); pattern B, low vascular density (mean CD31: 103 spots, SD: 57) (8% of cases); pattern C, mixed heterogeneous angiogenic signature (82% of cases). Similar patterns were observed on CD34 immunostaining with pattern A (mean CD34: 303 spots, SD: 108); pattern B (mean CD34: 163 spots, SD: 57) and pattern C, again characterized by mixed heterogeneous angiogenic signature.

These patterns were found to be identifiable by simple microscopy by the three coordinating pathologists (Guido Martignoni, Anna Caliò, Matteo Brunelli) with a concordance of 0.86 for pattern A, 0.56 for pattern B and 0.81 for pattern C.

Statistical analysis revealed concordance between immunostaining of CD31 and CD34 (k-Cohen: 0.8). Similar indices were obtained for vascular immunohistochemistry for *3D fusion* multisite sampling versus TMA cores, whereas poor concordance was obtained between values from routine sampling versus TMA cores or *3D fusion* multisite samples (k-Cohen: 0.3).

**Figure 5:**
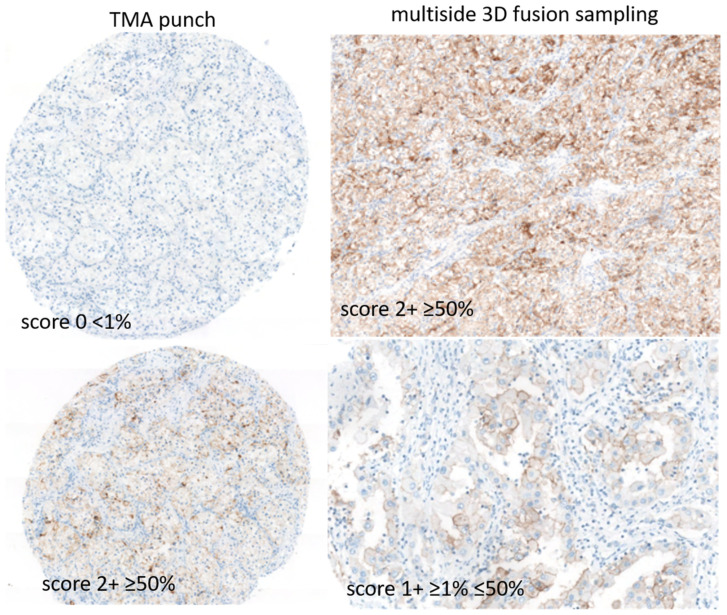
Classification of PD-L1 immune activation in clear cell RCCs after *3D fusion* multisite tumor sampling. Diagram showing categorization of immune patterns: score 2 strong expression; score 1, low/weak expression; score 0 with absence of any expression.

**Figure 6:**
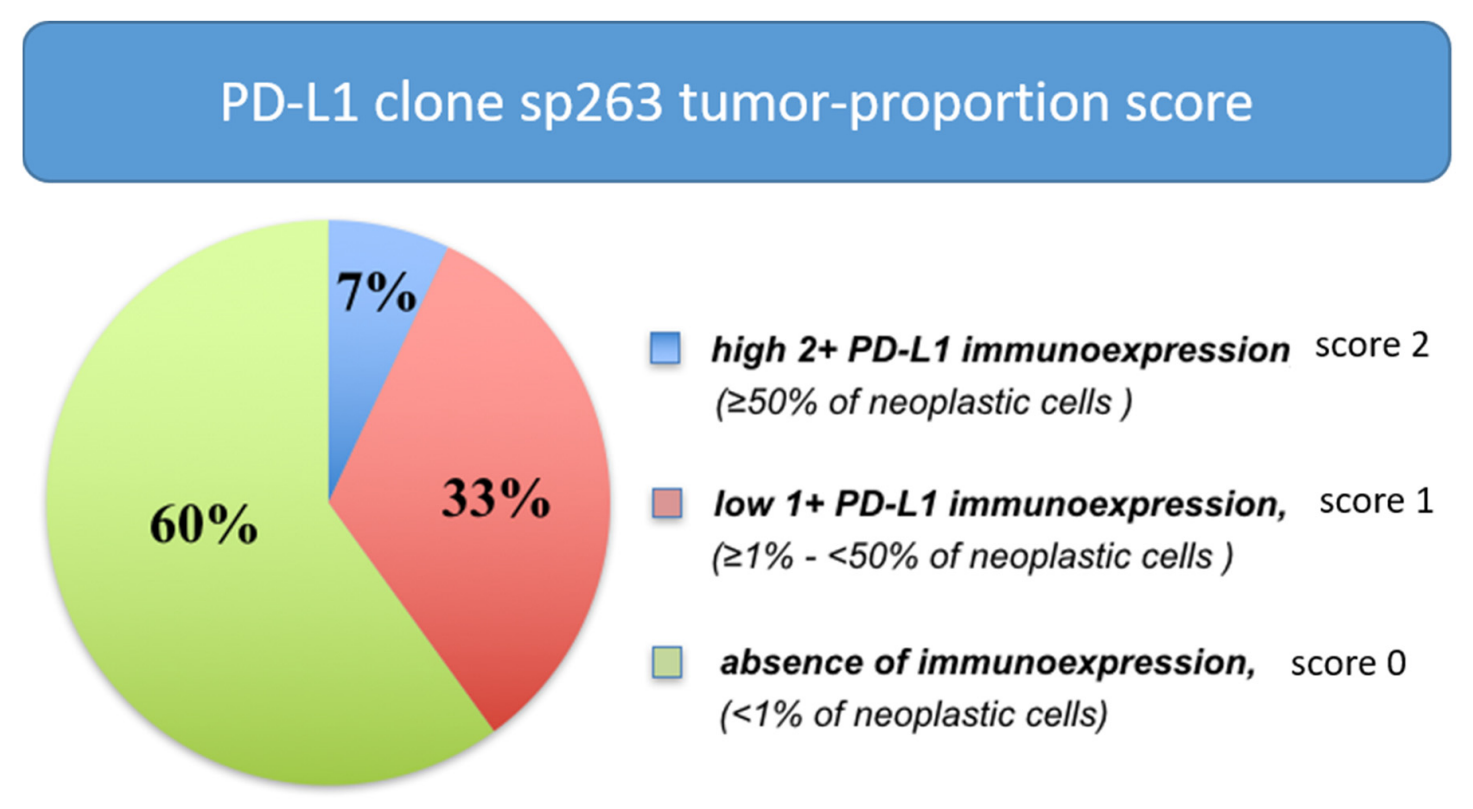
Distribution of PD-L1 immunohistochemical expression in clear cell RCCs after *3D fusion* multisite tumor sampling.

PD-L1 diffuse immune expression (≥50% of neoplastic cells) was seen in a minority of clearRCC (7%), a low level of PD-L1 expression (≥1%–<50% of cells) was observed in 33% of cases and absence of expression (<1%) in 60% of cases, after in toto 3D tumor inclusion (Figure 5 and Figure 6).

CD8 and PD-L1 expressions were higher and correlated (k-Cohen: 0.8) in the undifferentiated rhabdoid and sarcomatoid clearRCC subtypes. The same mesenchymal signature was observed for the four genes at the mRNA level (Figure 3), consistent with the undifferentiated nature of the subtypes.

Of note, 30% of score 1 cases set 0 at routine sample analysis. Both PD-L1 E1L3N and sp263 clones displayed concordance (k-Cohen: 0.82).

### 3.4. Evaluation of 3D Fusion Sections

Examination of the 3D fusion sections stained with CD31 and CD34 showed the vascular pattern to be homogenous (patterns A and B) in 18% of cases, with overlapping CD31 and CD34 staining. In 18% of these tumors, two patterns of homogenous angiogenesis were found. The first of these was a high level of angiogenesis, which was seen in 10% of tumors. The second pattern consisted of a low level of angiogenesis, which was present in 8% of tumors. The remaining 82% of cases were characterized by high levels of angiogenesis with adjacent zones showing low-density angiogenesis.

CD31/CD34 and CD8/PD-L1 comparisons revealed similar profiles between multisite 3D fusion and in toto tumor samples (*p* = 0.03), whereas discordances in standard sampling (*p* = 0.05). ISUP/WHO grading was upgraded in 26% of cases (G3–G4), and necrosis and sarcomatoid/rhabdoid characters were observed in 11 and 7% of cases, respectively, only after 3D fusion (both *p* = 0.03).

Again, 22% of cases were set to intermediate to high risk of clinical recurrence by using 3D fusion compared to standard methods (*p* = 0.04), mainly due to findings of all aggressive characters such as grading G4, presence of sarcomatoid/rhabdoid characters and tumoral necrosis.

## 4. Discussion

In this study, we showed that the *3D fusion* gross sampling method is simple to perform and negates the bias of heterogeneity in renal cell carcinoma (RCC) sampling and molecular profiling. Further, we demonstrated that the number of paraffin blocks required from multisite sampling, to overcome intratumoral heterogeneity, is equal to the maximum diameter of the neoplasms. For example, a tumor measuring 7 cm as maximum diameter requires seven blocks with multisite random sampling (six random tissue fragments per block), whereas a tumor measuring 9 cm as maximum diameter requires nine blocks, each containing six tissue fragments. Despite the identification of a number of prognostic parameters that have shown clinical utility for clearRCC, overall survival varies among patients, due primarily to the biological phenomenon of morphological and molecular heterogeneity [1]. Neovascularization is one of the biological processes that characterize clearRCC, and for this reason, angiogenic inhibitors are considered to be a first-line therapy for tumors that cannot be surgically cured [33]. While an understanding of the dynamics and morphology of angiogenesis may ultimately inform both immunotherapy and anti-angiogenic treatments, the efficacy of this may be hampered by observations producing skewed results resulting from inadequate sampling. Previous studies have stressed this point, noting that standard sampling protocols may be insufficient to negate tumoral heterogeneity, thus confounding treatment decisions and prognostic assessment [1,34]. The aim of our study was to develop a new approach to tumor gross sampling that is able to profile the entire tumor, and we applied this to the evaluation of angiogenesis in our series of clearRCC. As a component of the study, we also validated the use of a digital system to assess the number of vessels and vascular density, by comparing a standard manual count of vascular structures with digital analysis data.

Recently, adjuvant therapies have been proposed in locally advanced renal cancer, as pembrolizumab treatment has led to a significant improvement in disease-free survival compared with placebo after surgery among patients with kidney cancer who were at high risk for recurrence. A significant cohort of patients from the actual study does harbor characteristics that fit perfectly in this setting and does merit proposals for adjuvant therapies. The 3D fusion method increases assessment of advanced-stage patients after sampling (more G4 and more sarcomatoid/rhabdoid morphological characters with clinical relevance) [27]. A significant proportion of cases from our study increased the grading (26% from G3 to G4) and presence of necrosis (additional 11%). We believe these findings are clinically relevant in light of the recently approved new chances of therapies [27], due to the evidence that 22% of cases were set to intermediate to high risk of clinical recurrence by using 3D fusion compared to standard methods.

At the technical level, the extent to which the digital examination method and 3D fusion improve the result issued by the pathologist and the impact to which this report modifies the complementary oncological treatment is clear by the fact that such a methodology does harbor standardization of the analytical level and automation. The standardization brings quality to the appropriate selection of patients [35]. The proposed 3D fusion gross examination of renal carcinoma and the digital examination of histological and immunohistochemical slide evaluation have statistically significantly different results from that of the standard method (both *p* = 0.03). Along with this issue, we believe an AI/machine learning algorithm on multisite 3D fusion samples may be an attractive project in the near future.

Previous studies on the vascularity of clearRCCs have produced conflicting results. Many biomarkers are used for tissue diagnostics in tumors arising from the urogenital tract (for example, CAIX, S100A1, cathepsin-k, CK7, CD10 and others); however, difficulties have been observed among biomarkers for real neoangiogenesis grading versus the immunoenvironment [36,37].

It is clear from the detailed analysis of these results that there is a considerable variation in methodology. Some studies utilized mixed tumor groups that included clear cells and other types of renal neoplasia. In many of the studies that were confined to tumors with clear cell morphology, it is apparent that inadequate sampling is a major issue. In particular, for some studies, random archival sections were used, while in other studies, so-called hot spots of the highest tumor grade were chosen. In no study has there been a complete sampling of the tumor. A further point of concern relates to the way in which tumors were visualized. Recent reports have indicated that there appear to be two populations of vessels present that may be differentiated according to their respective immunoexpression. In this study, it was demonstrated that the vessels that have cells that are CD34 positive are more highly differentiated [16], while those that were CD31 positive and CD34 negative were classified as undifferentiated. This variation in staining means that if CD34 is utilized as the only vascular marker, as is the case in most published studies, then some vessels will be overlooked.

In our detailed analysis of the vascular tree associated with clearRCC, we showed three distinct patterns of vascular morphology. The first of these has a homogenous morphology consisting of a rich vascular network. The second vascular pattern is also homogenous, but here, the vascular density is much lower. The third pattern is distinctly heterologous in morphology, consisting of a rich network of small vessels intermixed with medium-sized vessels.

Our study demonstrated that a cut-off of CV < 0.2 with respect to MVD values can discriminate homogenous from heterogeneous vascularity, with homogenous morphology being seen in approximately one-fifth of cases. In the homogenous vessel group, absolute values of vessels/MVD were then able to discriminate tumors showing high and low levels of vascularity.

While this study has shown that 3D fusion multisite sampling of clearRCC negates problems associated with tumor undersampling, we acknowledge that in routine laboratory practice, it is not always possible to submit the tumor in toto. It is, however, apparent that the current recommendations that sampling be limited 1 block/cm of tumor tissue diameter are inadequate. We recommend that, for completeness of sampling and to negate the tumor heterogeneity that is characteristic of renal cell carcinoma, *3D fusion* sampling be performed as a routine procedure.

## Figures and Tables

**Table 1:** Clinicopathological findings in 100 advanced ≥pT3a staged clearRCC.

Males	69
females	31
right kidney	54
left kidney	46
pT3a	68
pT3b	28
pT4	6
grading sec. ISUP/WHO 2016	
G1	0
G2	9
G3	61
G4	30
rhabdoid differentiation	9
sarcomatoid differentiation	8
gross images collected	656
paraffin blocks embedded	5231
Tissue microarray array (TMA) sections	3093

**Table 2:** Neoangiogenesis measured as count of vessels in clearRCCs after fusion 3D multisite sampling (tumor included in toto).

	Size Samples	CD31	CD34	Vessels/mm^2^	
Routine sampling	one sample (3 × 1.5 cm) per cm single block	5–389	7–410	816	manual count
		13–408	21–478	899	digital count
Tissue microarray (TMAs) sampling	0.6 mm tissue core sample	41–480	49–393	432	manual count
				524	digital count
Fusion 3D multisite tissue sampling	six samples (0.5 × 0.5 cm) per cm single block	21–465	15–691	995	manual count
		19–510	32–680	1001	digital count

## Data Availability

Not applicable.
